# Antibacterial properties of *Acinetobacter baumannii*phage Abp1 endolysin (PlyAB1)

**DOI:** 10.1186/s12879-014-0681-2

**Published:** 2014-12-12

**Authors:** Guangtao Huang, Xiaodong Shen, Yali Gong, Zhiwei Dong, Xia Zhao, Wei Shen, Jing Wang, Fuquan Hu, Yizhi Peng

**Affiliations:** State Key Laboratory of Trauma, Burns and Combined Injury, Institute of Burn Research, Southwest Hospital, Third Military Medical University, Chongqing, China; Department of Microbiology, Third Military Medical University, Chongqing, China; Department of Biochemistry and Molecular Biology, Third Military Medical University, Chongqing, China

**Keywords:** Pandrug-resistant Acinetobacter baumannii, Endolysins, Multilocus sequence typing

## Abstract

**Background:**

*Acinetobacter baumannii* has emerged as one of the most important hospital-acquired pathogens in the world, because of its resistance to almost all available antibiotic drugs. Endolysins from phages are attracting increasing interest as potential antimicrobial agents, especially for drug-resistant bacteria. We previously isolated and characterized Abp1, a virulent phage targeting the multidrug-resistant *A. baumannii* strain, AB1.

**Methods:**

To evaluate the antimicrobial potential of endolysin from the Abp1 phage, the endolysin gene *plyAB1* was cloned and over-expressed in *Escherichia coli*, and the lytic activity of the recombinant protein (PlyAB1) was tested by turbidity assessment and bacteria counting assays.

**Results:**

PlyAB1 exhibits a marked lytic activity against *A. baumannii* AB1, as shown by a decrease in the number of live bacteria following treatment with the enzyme. Moreover, PlyAB1 displayed a highly specific lytic effect against all of the 48 hospital-derived pandrug-resistant *A. baumannii* isolates that were tested. These isolates were shown to belong to different ST clones by multilocus sequence typing.

**Conclusions:**

The results presented here show that PlyAB1 has potential as an antibiotic against drug-resistant *A. baumannii*.

**Electronic supplementary material:**

The online version of this article (doi:10.1186/s12879-014-0681-2) contains supplementary material, which is available to authorized users.

## Background

*Acinetobacter baumannii* is a Gram-negative, non-fermentative, nonmotile and oxidase-negative coccobacillus that usually exists in sewage, water, and healthcare facilities [1]. *A. baumannii* can cause nosocomial pneumonia, bacteremia, and infections of the urinary tract, skin, and soft tissues. *A. baumannii* has become the focus of significant attention worldwide, because of its ability to rapidly develop antibiotic resistance. This has led to the emergence of multidrug-resistant *A. baumannii* (MDRAB) and pandrug-resistant *A. baumannii* (PDRAB), within a few decades. PDRAB refers to isolates that are resistant to all available anti-*A. baumannii* antimicrobial agents, except for polymyxins [2]. In recent years, many outbreaks of *A. baumannii* infections have been reported. A significant nosocomial outbreak of MDRAB in France occurred in 2006–2008 in a burns unit at Saint-Antoine Hospital, and another occurred in the United States in a military treatment facility caring for injured service personnel and civilians during 2003–2005 [3].

*A. baumannii* is becoming a serious clinical problem, and is now receiving increasing attention from clinicians and pharmaceutical scientists. In the 1980s, many antibiotics could be used to treat *A. baumannii* infections. However, less and less antibiotics are effective now [1],[2],[4]. To date, polymyxin and tigecycline are the only effective antibiotics left to treat MDRAB infections. Worryingly, polymyxin- and tigecycline-resistant *A. baumannii* strains have been isolated in many places around the world [5]. We may face the unfortunate situation where we may have no antibiotics available for treating *A. baumannii* infections in the future.

Endolysins are peptidoglycan hydrolases encoded by bacteriophages that are released at the terminal stage of the replication circle to degrade the peptidoglycan bacterial wall from within [6]. As a result, endolysins are attracting increasing interest as potential antimicrobial agents, especially for multidrug-resistant bacteria [7]. Many studies have shown the antibacterial potential of endolysin, partly through animal models [7],[8]. PRF-119, as a recombinant chimeric bacteriophage endolysin, exhibited very high antibacterial activity, specifically against *Staphylococcus aureus*. Doehn *et al*. [9] showed that delivery of the endolysin Cpl-1 by inhalation could reduce mortality in mice with fatal pneumococcal pneumonia by 80%.

In a previous study, we isolated and characterized Abp1, a virulent phage targeting the MDRAB strain, AB1 [10]. We found that phage Abp1 could produce large, clear plaques on the lawn of its host, implying that Abp1 endolysin has strong lytic activity. In the present study, we cloned and over-expressed the endolysin-encoding gene, and determined the lytic activity of the recombinant protein, PlyAB1. Through a series of functional assays, we have shown that PlyAB1 exhibited significant antibacterial activity against all 48 PDRAB isolates collected from the Southwest Hospital of Chongqing, China. These results indicate the potential use of PlyAB1 as an alternative antibacterial agent against drug-resistant *A. baumannii* infections.

## Methods

### Bacteria, plasmids, and growth conditions

The bacterial strains, plasmids, and primers used in this study are listed in Table 1. AB1 is a clinical *A. baumannii* isolate from the blood of a patient with severe burns, and Abp1 is a lytic phage of AB1. The clinical *A. baumannii* isolates, AB01–AB48, are from the Southwest Hospital of Chongqing, China. *Escherichia coli* strains JM109 and BL21, *Staphylococcus aureus* strain N315, and *Pseudomonas aeruginosa* strain PAO1 are kept in our laboratory. All bacteria were cultured in Luria–Bertani (LB) broth or agar at 37°C.

**Table 1:** **Primers, phage, bacterial strains, and plasmids used in this study**

Name	Characteristics / function	Source
P1	GGATCCATGATTCTGACTAAAGACGGGTT	Beijing Genomics Institution
P2	CTCGAGCTATAAGCTCCGTAGAGCGC	Beijing Genomics Institution
OXA-51-F	TAATGCTTTGATCGGCCTTG	Beijing Genomics Institution
OXA-51-R	TGGATTGCACTTCATCTTGG	Beijing Genomics Institution
BL21(DE3)	Expression host for recombinant plasmid	Purchased from Sangon Biotech
pET28a	Expression vector (kanamycin resistant)	Our laboratory collection
pET28a-*plyAB1*	Recombinant vector (kanamycin resistant)	This study
Abp1	Phage of AB1. Isolated from hospital sewage	Our laboratory collection
AB001-AB040	XDRAB. Isolated from the Southwest Hospital of Chongqing. China	Our laboratory collection
JM109	*E. coli* strain for testing host range	Our laboratory collection
BL21	*E. coli* strain for testing host range	Our laboratory collection
N315	*S. aureus* strain for testing host range	Our laboratory collection
PAO1	*P. aeruginosa* strain for testing host range	Our laboratory collection

### Identification and cloning of the putative endolysin from Abp1

Sequence alignments were performed using the basic local alignment search tool at the National Center for Biotechnology information (http://www.ncbi.nlm.nih.gov/BLAST/). Protein domain and tertiary structure analysis were conducted using Pfam (http://pfam.xfam.org/) and Swiss-Model (http://swissmodel.expasy.org) online servers. Genomic DNA from phage Abp1 was extracted using previously described methods [10]. The Abp1 endolysin-encoding gene was amplified with P1 and P2 primers (Table 1) and PrimSTAR® HS DNA Polymerase (TaKaRa, Japan). PCR products were purified using a Wizard SV Gel kit (Promega, USA). After digesting with *Bam*HI and *Xho*l restriction enzymes (Fermentas, USA), the DNA fragment of interest was cloned into the corresponding sites of the pET28a expression vector. Enzyme analysis and DNA sequencing were conducted to confirm the successful construction of the recombinant expression plasmid, pET28a-*plyAB1*, which was transformed into *E. coli* BL21 (DE3).

### Over-expression and purification of PlyAB1

*E. coli* BL21 (DE3) cells harboring the recombinant plasmid pET28a-*plyAB1* were grown overnight in LB medium containing kanamycin (50 μg/ml, Sangon Biotech, China). An overnight culture of cells was diluted 100-fold in 300 ml of LB medium and then incubated at 37°C until the OD_600_ reached 0.6. To induce expression of the target protein, a final concentration of 1 mM isopropyl β-D-1-thiogalactopyranoside (IPTG) was added to the culture followed by incubation at 16°C for 20 hours. Cells were harvested by centrifugation and resuspended in 30 ml of Hepes/KOH buffer (pH 7.4, 20 mM Hepes/KOH, 140 mM NaCl, 1% Triton X-100) [11]. The cell lysate was disrupted further by freeze-thawing cycles (−20°C alternating with room temperature) and sonication (5 s pulse, 20 s rest over 30 min). After centrifugation and filtration, the supernatant was loaded onto a Ni-nitrilotriacetic acid column (Ni-NTA; Qiagen, Germany) and a washing buffer (0.5 M NaCl, 20 mM Tris–HCl, pH 7.4) was applied with increasing imidazole concentrations (20 mM, 50 mM, 100 mM, 200 mM, 500 mM). The eluted protein of interest was enriched by Amicon Ultra-15 (Millipore, USA) filtration and the concentration was determined by the Lowry method. The purity of the recombinant endolysin was analyzed by sodium dodecyl sulfate polyacrylamide gel electrophoresis (SDS-PAGE). N-terminal amino acid sequencing was performed to verify the identity of the purified protein.

### Lytic activity of recombinant PlyAB1 against AB1

AB1 was grown to exponential phase, and 1 ml of culture was centrifuged at 16,000 × g for 2 min. The cell pellets were suspended in 500 μl of Hepes/KOH buffer. The lytic assay was performed in a 96-well plate and analyzed in a microplate reader (SpectraMax M2e, Molecular Devices) using SoftMax Pro. Suspended bacteria (158 μl) were added to each well, and 42 μl of the purified PlyAB1 protein (2.4 mg/ml) in the trial group or the same volume of reaction buffer (0.5 M NaCl, 20 mM Tris–HCl, pH 7.4) in the negative control group was added. The parameters used by SoftMax Pro were as follows: read interval 60 s, shake 40 s, temperature 37°C, total time 30 min. Changes in the OD_600_ values were recorded.

Cell pellets from 1-ml AB1 cultures at logarithmic phase were collected though centrifugation (16,000 × g, 2 min). To remove the outer cell membrane, the AB1 cell pellets were suspended in 500 μl of Hepes/KOH buffer. A 20 μl volume of the recombinant PlyAB1 protein (1 mg/ml) was added to 180 μl of cell suspension in a 1.5-ml microcentrifuge tube. At the beginning of the experiment and 30 min later, the number of viable bacteria (colony forming units; CFU) was assessed by counting the colonies formed [12].

### Collection and characterization of PDRAB isolates

A total of 48 clinical isolates (see Additional files 1 and 2) of A. *baumannii* were collected from the Southwest Hospital of China from January 2012 to January 2014 (all the isolates were collected as part of standard patient care and informed content of the patients or their families was obtained). All 48 isolates were phenotypically identified to genus level by the API 20-NE system (Biomerieux, France). Genotypic identification was performed by PCR detection of the intrinsic *bla*_OXA-51-like_ gene [13] and sequence analysis of the 16S rRNA gene [14]. The antimicrobial susceptibility of these isolates was determined by the K-B method and the results were interpreted according to the Clinical and Laboratory Standards Institute (CLSI) criteria (http://clsi.org/). Multilocus sequence typing (MLST) was used to investigate the molecular epidemiology of the isolates with seven housekeeping genes comprising *gltA*, *gyrB*, *ghdB*, *recA*, *cpn60*, *gpi*, *and rpoD*. The primer sequences, PCR reaction conditions and MLST analysis that were used have all been previously described [15].

### Lytic activity of PlyAB1 against clinical PDRAB isolates

The lytic activity of the recombinant PlyAB1 protein against the 48 PDRAB clinical isolates were determined by a microplate reader and directly with photography, as described above.

### Determination of the lytic range of PlyAB1

To test the lytic range of PlyAB1, the lytic activity of the purified recombinant protein against *E. coli* (BL21 and JM109), *P. aeruginosa* (PAO1), and *S. aureus* (N315) were measured with a microplate reader, as described above. *A. baumannii* strain AB1 and reaction buffer were used as positive and negative controls, respectively. Changes in turbidity were also recorded directly using spectrophotometric measurement.

### Statistical analysis

Data were analyzed by a Student’s *t*-test and a value of *P* < 0.05 was considered significant.

### Ethical approval

Not required.

## Results

### Identification and characterization of *plyAB1*

**Figure 1:**
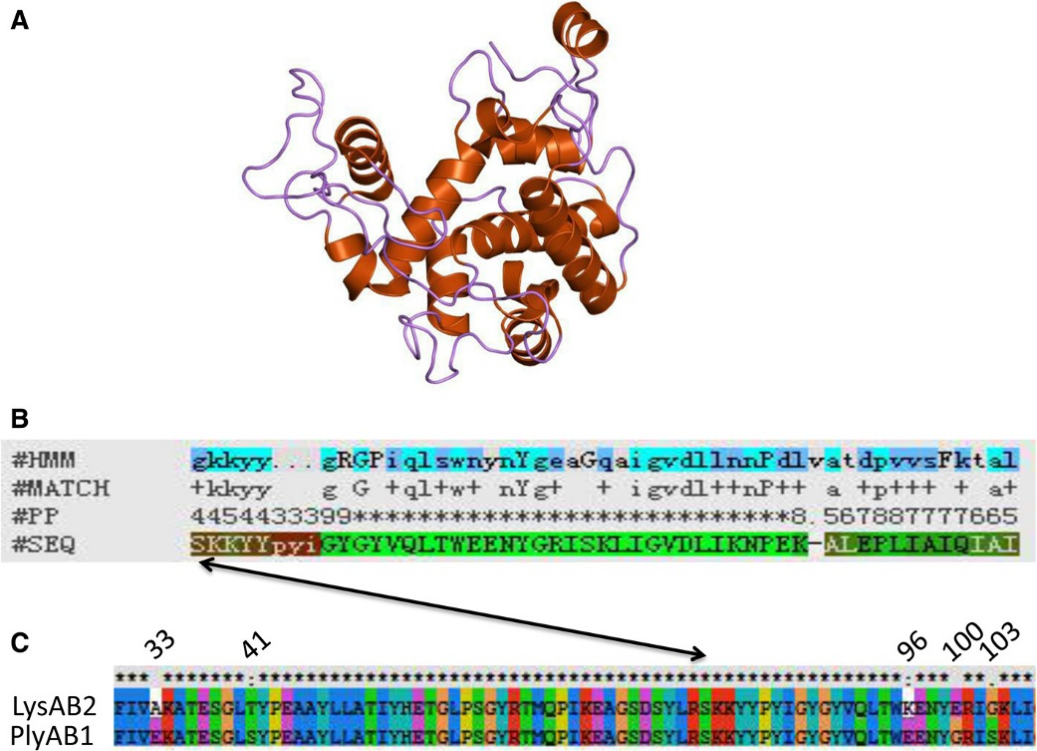
**Sequence analysis of PlyAB1. (A)** Locations of five mutations between PlyAB1 and LysAB2 shown by sequence alignment. **(B)** Analysis of the tertiary structure of PlyAB1 from the online SWISS-MODEL server. Five mutants are shown in the model: amino acids 33 (yellow), 41 (orange), 96 (green), 100 (red), and 103 (blue). **(C)** Visualization of the conserved domain (magenta) of PlyAB1. Three mutations (amino acids 96, 100, and 103) are located in the conserved domain.

ORF50 of the *A. baumannii* phage Abp1 was predicted to be the putative endolysin by Blastp. The Pfam database reveals that it contains a conserved domain known as glycoside hydrolase family 19. Glycoside hydrolase family 19 comprises enzymes with only one known activity, that is, catalyzing the hydrolysis of beta-1,4-N-acetyl-D-glucosamine linkages [16]. We named the *orf50* gene of Abp1, *plyAB1.* PlyAB1 encodes a 185 amino acid protein, of which amino acid positions 79 to 128 comprise the conserved domain. In spite of the high sequence similarity between PlyAB1 and LysAB2 [17], five mutations exist between them, three of which are located in the conserved domain (Figure 1).

### Cloning, over-expression and purification of PlyAB1

**Figure 2:**
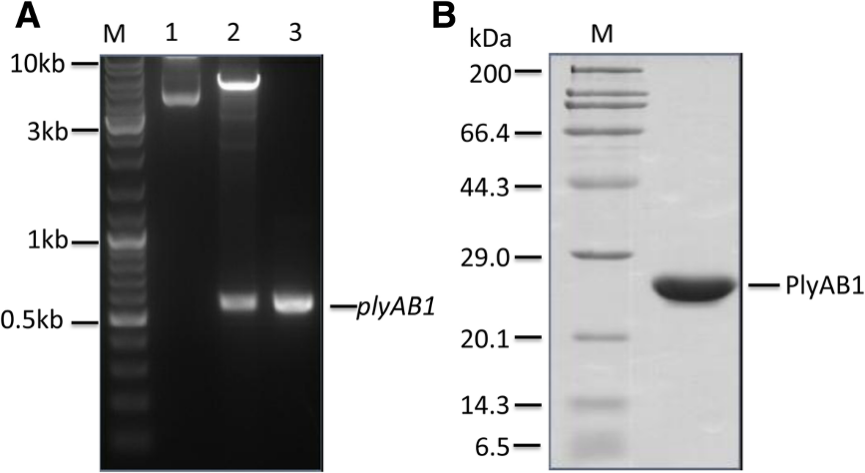
**Cloning and over-expression of PlyAB1. (A)** Cloning of *plyAB1* into pET28a: 1) recombinant plasmid pET28a*-plyAB1*, 2) pET28a*-plyAB1* digested with *Bam*HI and *Xho*l, 3) PCR amplification of *plyAB1*. **(B)** The purified PlyAB1 protein after Ni^+^ affinity chromatography and filtration.

The *plyAB1* gene was successfully amplified from Abp1 genomic DNA using the P1/P2 primer pair, and was then cloned into the *Bam*HI/*Xho*l sites of the pET28a expression vector. The recombinant plasmid generated (pET28a-*plyAB1*) was confirmed by enzyme digestion (Figure 2A) and DNA sequencing.

After induction with IPTG at low temperature (16°C), expression of the fusion protein, PlyAB1, with a predicted molecular size of 21 kDa, was detected by SDS-PAGE. We found that the recombinant protein was present mainly in the soluble fraction after sonication. The highly purified protein of interest was prepared by Ni-NTA affinity chromatography, and SDS-PAGE analysis indicated that a purity of more than 95% was achieved through use of the Quantity One software package (Bio-Rad) (Figure 2B). N-terminal peptide sequencing confirmed that the product we prepared was PlyAB1 (data not shown).

### PlyAB1 exhibited a strong lytic activity against AB1

**Figure 3:**
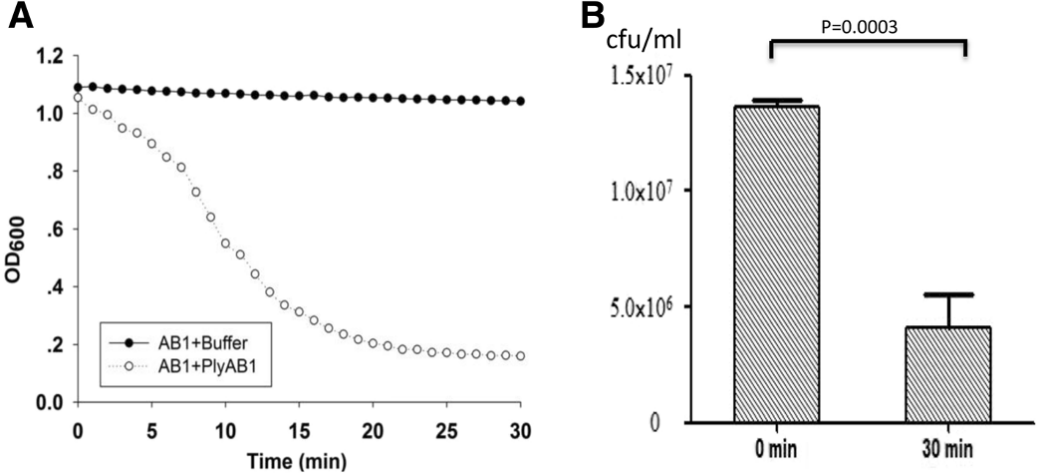
**Lytic activity of PlyAB1 against AB1. (A)** Lytic activity was determined by OD_600_ measurements. **(B)** The concentration of live bacteria decreased from 1.4 × 10^7^ CFU/ml at 0 min to 4.1 × 10^6^ CFU/ml at 30 min.

In Hepes/KOH buffer, *A. baumannii* AB1 was lysed effectively by the purified PlyAB1 protein. Within 30 min, the OD_600_ of the reaction between AB1 and PlyAB1 fell from 1.05 to 0.2 (Figure 3A). However, in the negative control group, the OD_600_ values of the AB1 and reaction buffer mixture showed almost no reduction. The viable bacterial counts results showed that the number of *A. baumannii* AB1 decreased from 1.4 × 10^7^ CFU/ml to 4.1 × 10^6^ CFU/ml in 30 minutes with 100 μg/ml of PlyAB1 (Figure 3B). The viable bacterial counts decreased by 82.6%.

### Characteristics of the clinical PDRAB isolates

**Figure 4:**
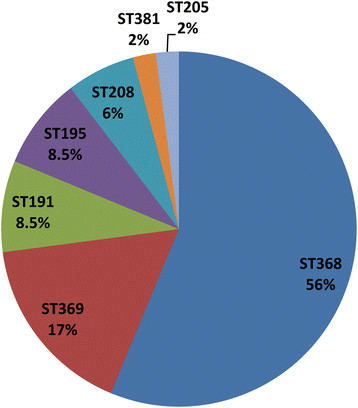
**Characterization of clinical pandrug-resistant** ***A. baumannii*** **isolates (PDRAB).** The 48 PDRAB isolates are grouped into seven different MLST types.

The *bla*_OXA-51-like_ gene is a species-specific gene of *A. baumannii* and was used here for identification. The 48 *A. baumannii* isolates were all found to harbor this gene*.* Of the 19 antibiotics tested, all 48 of the clinical strains were only sensitive to polymyxin B. However, they showed general resistance to cephalosporins, carbapenems, tetracyclines, aminoglycosides, sulfonamides, and quinolones. By definition, this shows all of the clinical *A. baumannii* strains are PDRAB. The PDRAB isolates could be grouped into seven existing MLST types (Figure 4). ST368 dominated, while 27 isolates belonged to ST368. Other MLST types included ST369 (8), ST191 (4), ST195 (4), ST208 (3), ST381 (1), and ST205 (1).

### Lytic activity of PlyAB1 against clinical PDRAB isolates

**Figure 5:**
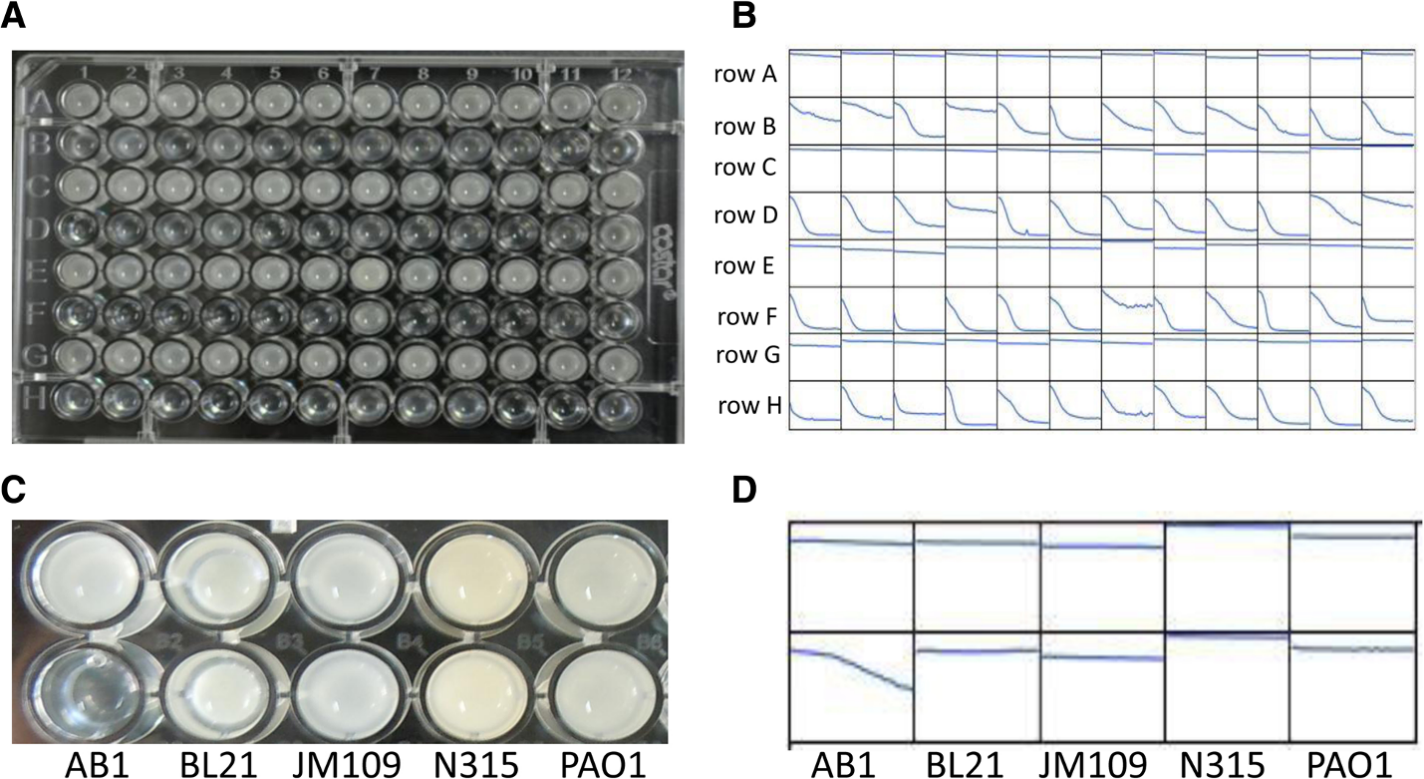
**Lytic activity of PlyAB1 against the 48 clinical PDRAB isolates. (A)** PlyAB1 lytic activity results for 48 PDRAB isolates 30 min after addition of the enzyme. Blue bar denotes the OD_600_ at 0 min, red bar denotes the OD_600_ 30 min later. **(B)** Rows A, C, E, and G represent the negative control groups containing buffer lacking PlyAB1 protein. Rows B, D, F, and H represent the trial groups (with PlyAB1 protein). Changes in the OD_600_ at 30 min were recorded by a microplate reader. **(C** and **D)** Lytic range of PlyAB1. Left to right: AB1, BL21, JM109, N315, and PAO1. PlyAB1 can only lyse *A. baumannii* AB1.

In the lytic activity assay, PlyAB1 digested all 48 of the hospital-derived PDRAB isolates (Figure 5A, B). As shown in Figure 5A, all the OD_600_ values of the PDRAB isolates and PlyAB1 decreased after 30 min incubation. The OD_600_ of the PDRAB isolates dropped significantly when purified PlyAB1 was incubated with them (Figure 5B, rows B, D, F, and H). For the negative control, the cell densities of the PDRAB isolates incubated with the same volume of reaction buffer as was used for the test samples did not change significantly (Figure 5B, row A, C, E, and G). In an even broader test, we found that PlyAB1 could rapidly lyse 206 of 212 additional clinical MDRAB isolates in 30 minutes (see Additional files 2 and 3).

### Lytic range of PlyAB1

To test the lytic range of PlyAB1, we used Gram-positive bacteria (*S. aureus* strain N315) and Gram-negative bacteria (*E. coli* strains BL21 and JM109 and the *P. aeruginosa* strain PAO1) as substrates. We found that PlyAB1 only lysed *A. baumannii*. It did not exhibit lytic activity against the *E. coli and P. aeruginosa* strains, or against the Gram-positive *S. aureus* strain (Figure 5C, D)*.* This result is consistent with previous reports that phage endolysin possesses specificity at the host level [6].

## Discussion

*A. baumannii* has emerged as an important nosocomial pathogen in various countries around the world [1]. This bacterium is known for its involvement in disease outbreaks in hospital and has the ability to spread in healthcare facilities [18]. *A. baumannii* infections have become more difficult to treat because of the emergence of isolates that are resistant to multiple antimicrobial drugs. Endolysin, as a peptidoglycan hydrolase, has attracted the attention of researchers as a potential antibiotic. To date, there are three endolysins known to target *A. baumannii* [17],[19],[20]. In the present study, we identified and characterized a new phage endolysin with activity against *A. baumannii*. We found it could not only degrade its host strain (AB1), but was also able to lyse all the clinical PDRAB isolates collected from the Southwest Hospital of Chongqing.

The results of the turbidity reduction assay and the bacterial counting assay revealed that recombinant PlyAB1 protein was able to lyse AB1. However, PlyAB1 could not degrade the cell walls of the other Gram-negative and Gram-positive bacteria that we tested. PlyAB1 protein showed relatively higher specificity for *A. baumannii* than it did for the other bacteria tested herein, a finding that differs from that of LysAB2, an *A. baumannii* phage endolysin that is active against Gram-positive and Gram-negative bacteria [17]. From a patient safety perspective, the high target-species specificity of endolysins is highly desirable. These enzymes specifically destroy their target pathogen without affecting the commensal microflora [7]. Although PlyAB1 has specificity at the species level, it showed relatively higher lytic activity against *A. baumannii* than did LysAB2. The OD_600_ values of the host strain decreased from 1.0 to about 0.65 with a concentration of 500 μg/ml of LysAB2 in 60 min, while the OD_600_ decreased from 1.05 to about 0.2 with same concentration of PlyAB1 within 30 min. Sequence alignments indicate that there are five mutations between these two proteins, and these may contribute to the increased lytic activity of PlyAB1. These mutations may change the net charge of the protein, which is known to improve the enzymatic efficiency of endolysins [21],[22].

It is known that endolysins can destroy the cell wall of Gram-positive bacteria by direct addition of lysins administered externally, but Gram-negative bacteria are naturally protected from endolysins by their outer membrane. This membrane prevents the penetration of lytic enzymes and protects the substrate, peptidoglycan, thus preventing lysis of Gram-negative cells; however, there are a few exceptions to this principle. It has been suggested that lysins from Gram-negative bacteriophages can lyse bacterial cells via mechanisms other than their enzyme activity. One study showed that lysins from Gram-negative bacteriophages can lyse both Gram-positive and Gram-negative bacteria when the outer membrane is removed [17]. Recently, genetically engineered endolysin-based artilysins have been shown to be effective at combating outer-membrane-intact multidrug resistant Gram-negative pathogens [23].

The PDRAB isolates were collected over 2 years, and they belong to seven different MLSTs. MLST is an unambiguous typing method that has achieved notable success in global epidemiological investigations [24]. Ruan *et al*. confirmed that ST92 was the most prevalent clone of carbapenem-resistant *A. baumannii* in China between 2009 and 2010 [13]. In our study, we found ST368 to be the most dominate clone, but ST92 was not detected. However, ST368 is a single-locus variant of ST92. They share six of seven allele numbers, with the *gpi* locus number differing between them.

As a murein hydrolase, PlyAB1 was able to degrade all 48 clinical PDRAB isolates within a relatively short time frame. Interestingly, we found that a few *A. baumannii* isolates were resistant to endolysin from the same species of bacteriophage that targets this bacterium (i.e., we found that PlyAB1 did not effectively lyse 6 of the 212 additional clinical MDRAB isolates that we tested). The reason for this may relate to potential differences in the peptidoglycan of PlyAB1-sensitive *A. baumannii* strains and that of PlyAB1-resistant strains [25],[26].

## Conclusions

Taken together, these results support the conclusion that PlyAB1 has potential as an antibiotic against drug-resistant *A. baumannii*. With an estimated 10^31^ phages worldwide, phage-encoded endolysins could be a vast resource for exploiting antibacterial agents against pathogens.

## Additional files

## Electronic supplementary material

Additional file 1: **Detail information of 48 pandrug-resistant** ***A. baumannii*** **isolates.** AMK = amikacin; SAM = ampicllin/sulbactam; POL = polymyxin B; SXT = sulphamethoxazole trimethoprim; CIP = ciprofloxacin; MEM = meropenem; MNO = minocycline; NET = netilmicin; GEN = gentamicin; TCY = tetracycline; CAZ = ceftazidime; FEP = cefepime; CSL = sulbactam/cefoperazone; CTX = cefotaxime; TOB = tobramycin; IMP = imipenem; LVX = levofloxacin; TZP = piperacillin/tazobactam. (XLS 2 MB)

Additional file 2: **Lytic activity of PlyAB1 on other clinical A. baumannii isolates.** Figure S1-S5, Lytic activity of PlyAB1 on other 212 clinical *A. baumannii* isolates. Red triangle indicates the PlyAB1-resistant strain. Figure S6-S7, rep-PCR of 48 pandrug-resistant *A. baumanni*i isolates. (PDF 448 KB)

Additional file 3: **Information of other 212 clinical** ***A. baumannii*** **isolates.** AMK = amikacin; SAM = ampicllin/sulbactam; POL = polymyxin B; SXT = sulphamethoxazole trimethoprim; CIP = ciprofloxacin; MEM = meropenem; MNO = minocycline; NET = netilmicin; GEN = gentamicin; TCY = tetracycline; CAZ = ceftazidime; FEP = cefepime; CSL = sulbactam/cefoperazone; CTX = cefotaxime; TOB = tobramycin; IMP = imipenem; LVX = levofloxacin; TZP = piperacillin/tazobactam. (XLS 58 KB)

Below are the links to the authors’ original submitted files for images.

Authors’ original file for figure 1

Authors’ original file for figure 2

Authors’ original file for figure 3

Authors’ original file for figure 4

Authors’ original file for figure 5

## Abbreviations

MDRAB: Multidrug-resistant *A. baumannii*
PDRAB: Pandrug-resistant *A. baumannii*
MLST: Multilocus sequence typing
CLSI: Clinical and Laboratory Standards Institute
