# Supplementary material for: Antibacterial properties of Acinetobacter baumanniiphage Abp1 endolysin (PlyAB1)
Source: BMC Infect Dis. 2014 Dec 12;14:681. doi: 10.1186/s12879-014-0681-2 (PMC4274762; doi:10.1186/s12879-014-0681-2)
Supplement: Supplementary file 2 — Additional file 2: Lytic activity of PlyAB1 on other clinical A. baumannii isolates. Figure S1-S5, Lytic activity of PlyAB1 on other 212 clinical A. baumannii isolates. Red triangle indicates the PlyAB1-resistant strain. Figure S6-S7, rep-PCR of 48 pandrug-resistant A. baumannii isolates. (PDF 448 KB) [file 12879_2014_681_MOESM2_ESM.pdf]

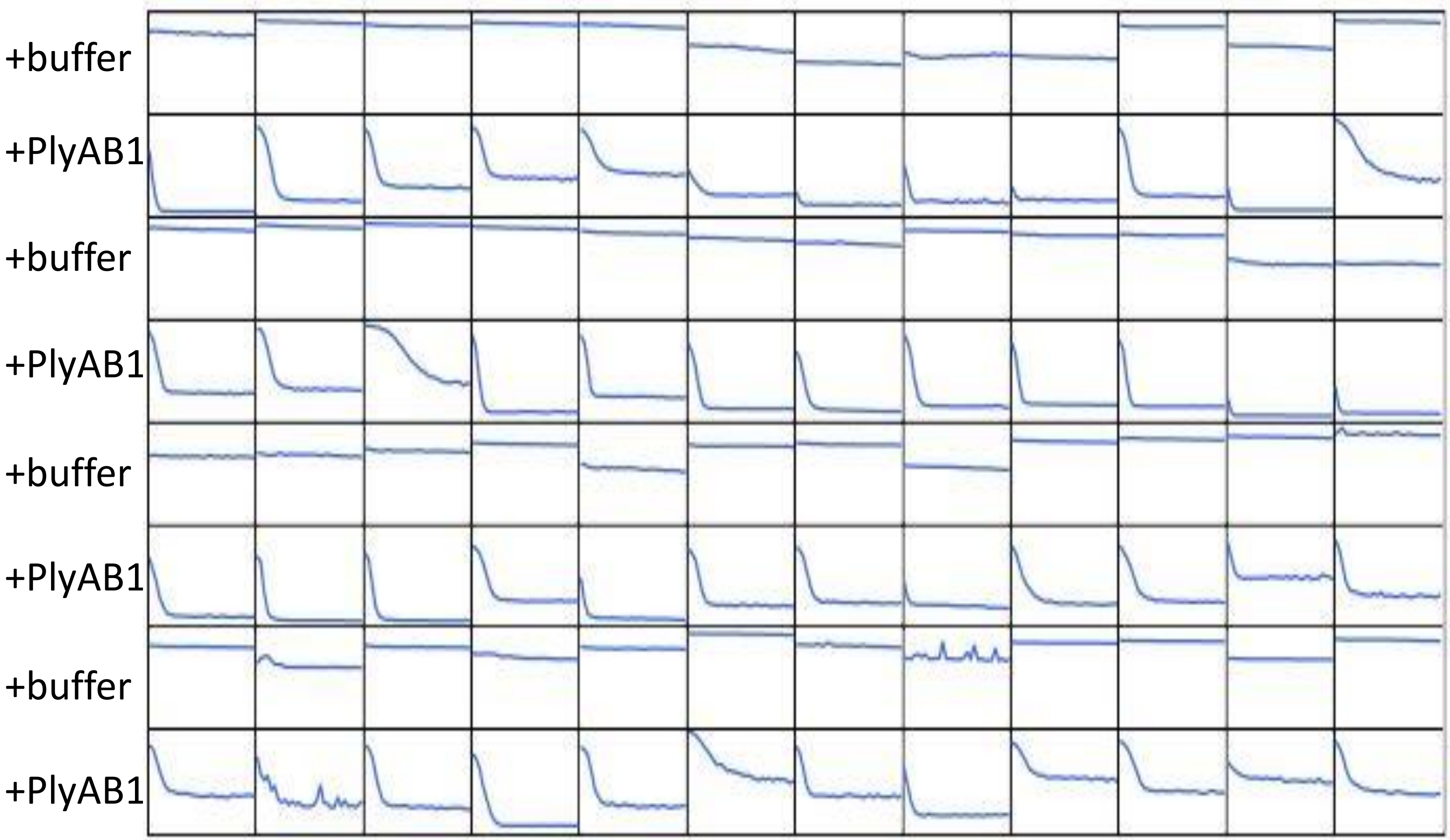

Fig,. S1 Lytic activity of PlyAB1 on clinical *A . Baumannii* isolates CQ1-CQ48

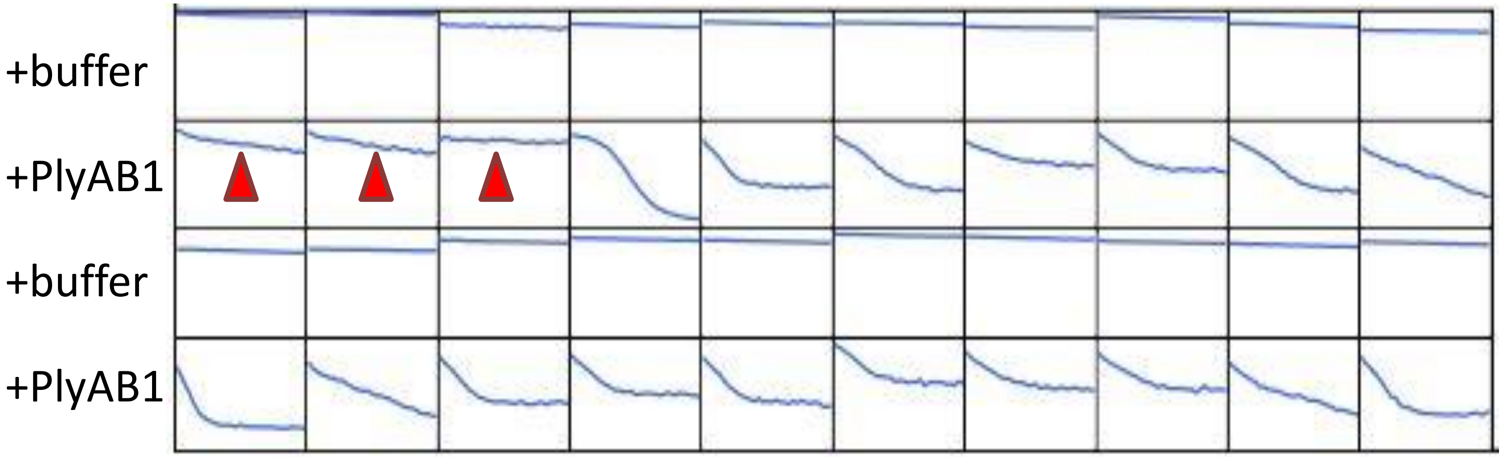

Fig,. S2 Lytic activity of PlyAB1 on clinical *A . Baumannii* isolates CQ49-CQ68, red triangle indicates the PlyAB1-resistant strain.

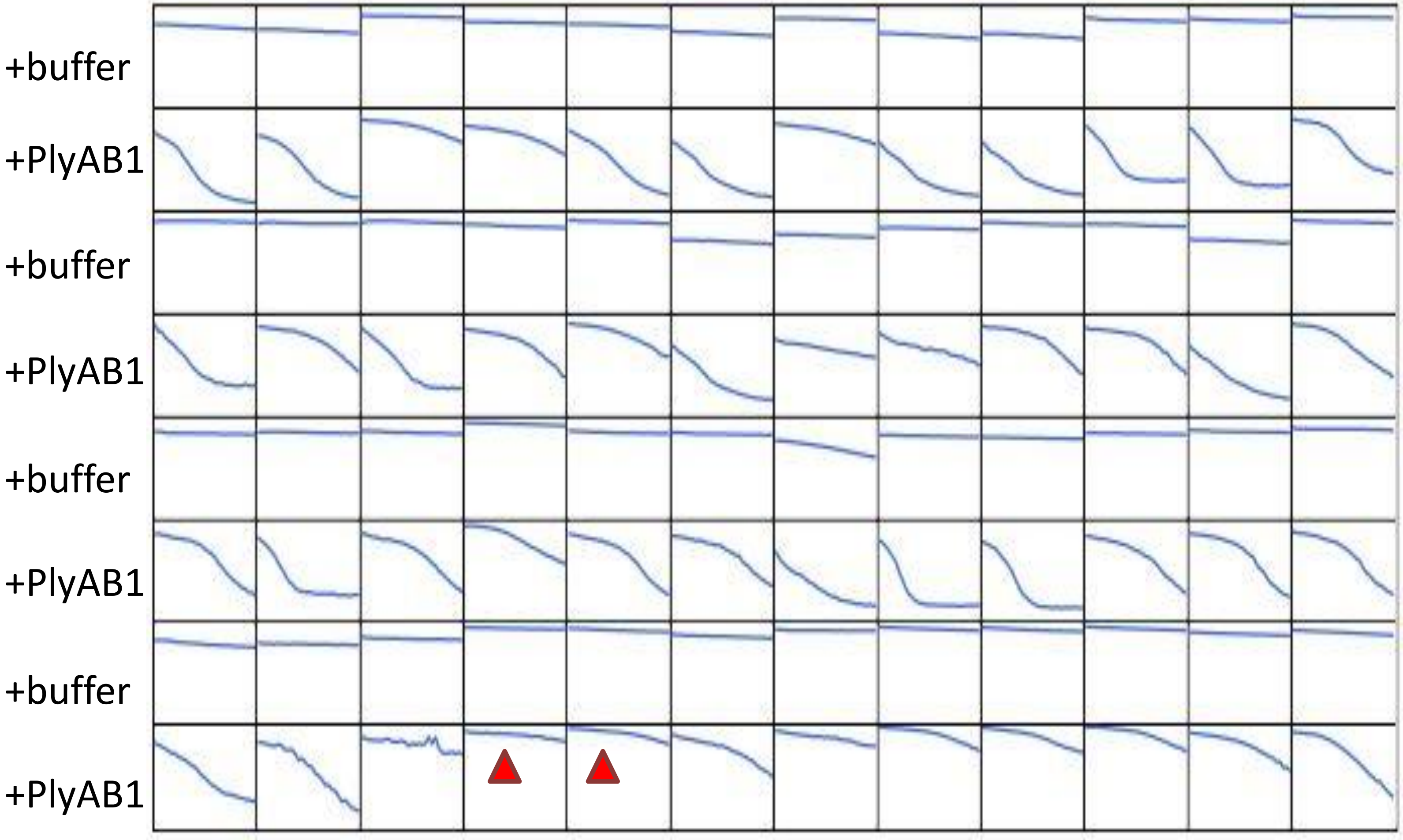

Fig. S3 Lytic activity of PlyAB1 on clinical *A . Baumannii* isolates CQ69-CQ116, red triangle indicates the PlyAB1-resistant strain

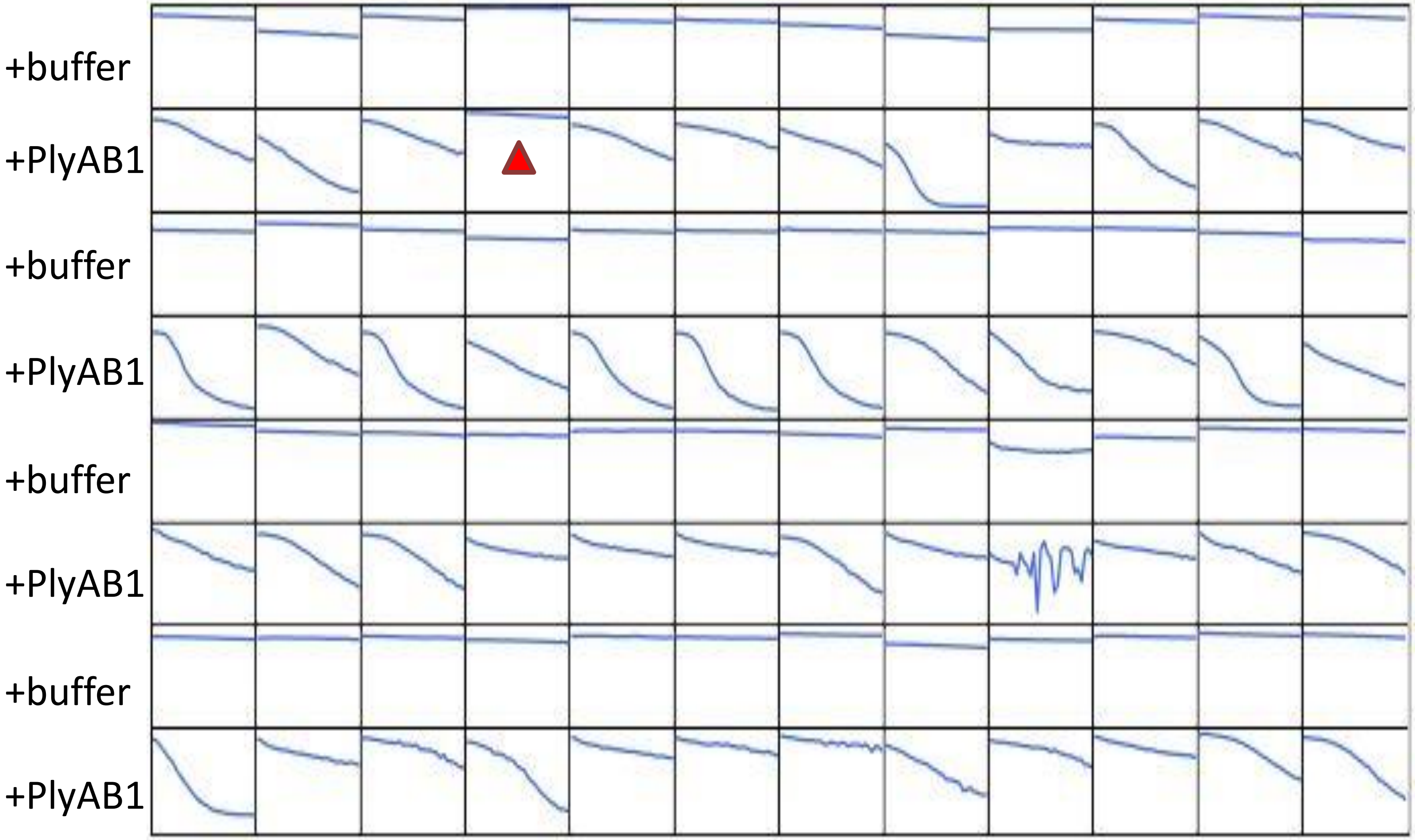

Fig. S4 Lytic activity of PlyAB1 on clinical *A . Baumannii* isolates CQ117-CQ164 , red triangle indicates the PlyAB1-resistant strain

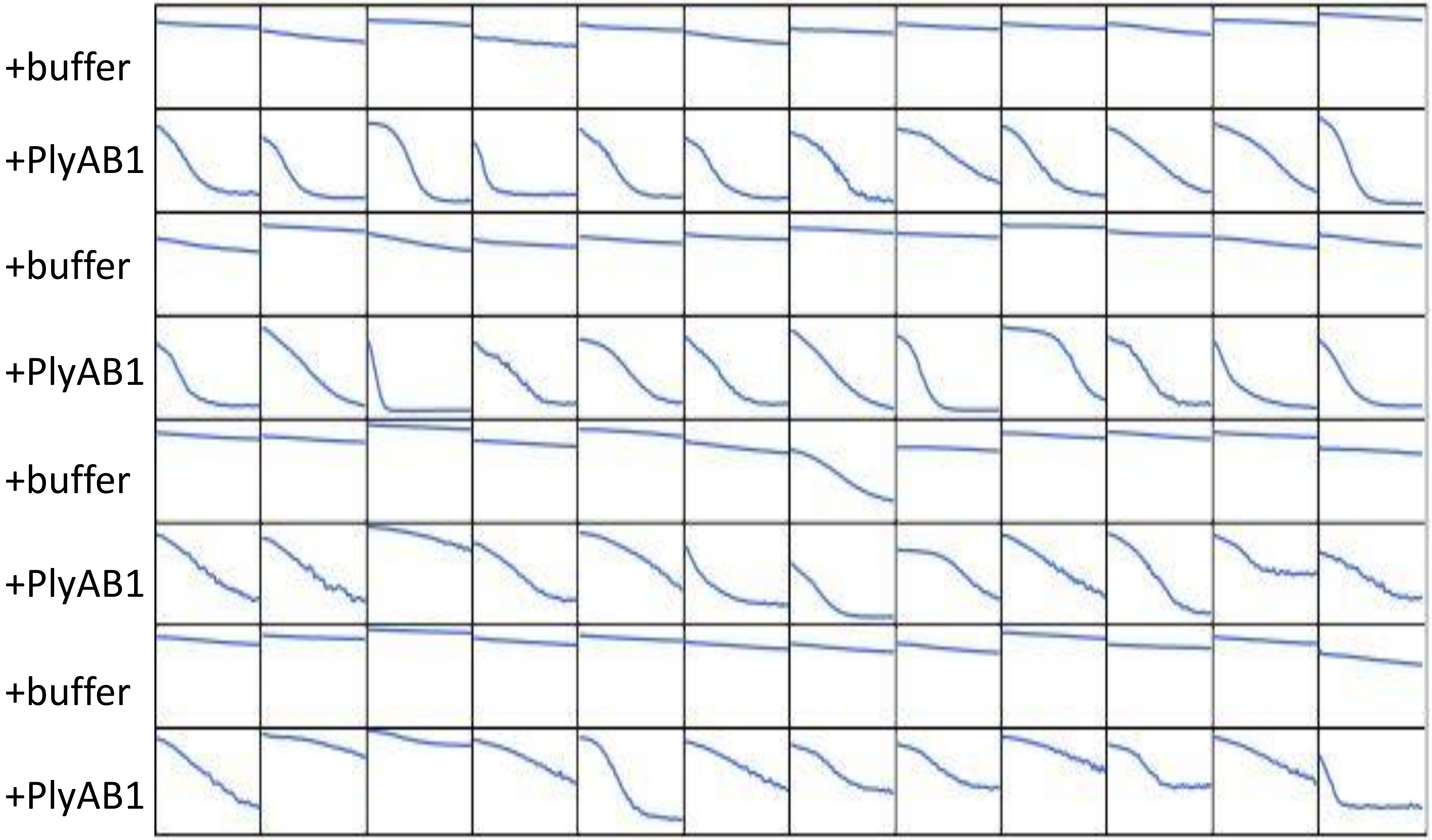

Fig. S5 Lytic activity of PlyAB1 on clinical *A . Baumannii* isolates CQ165-CQ212

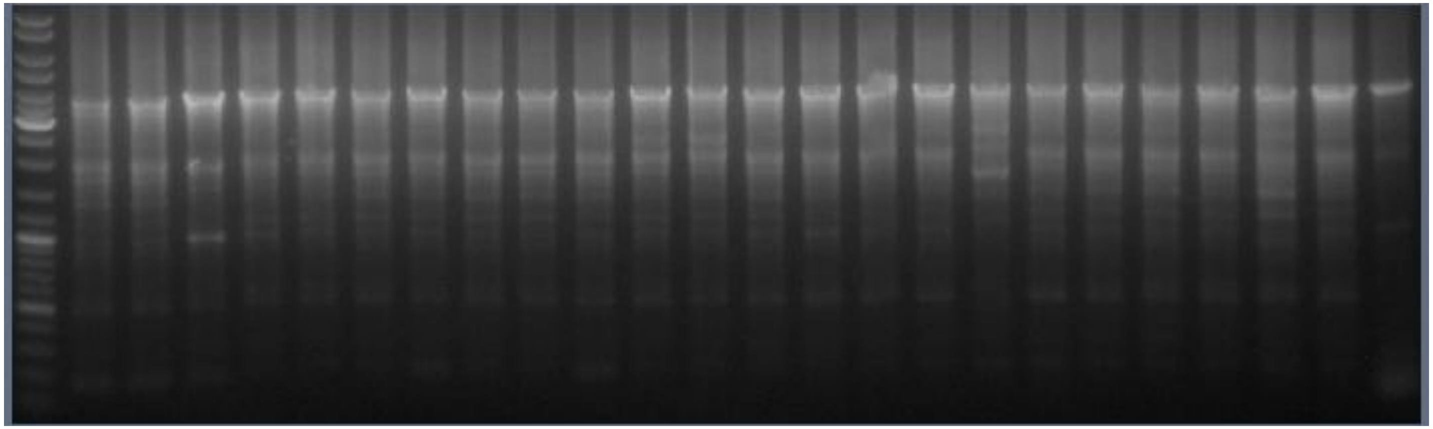

Rep-PCR AB01-AB24

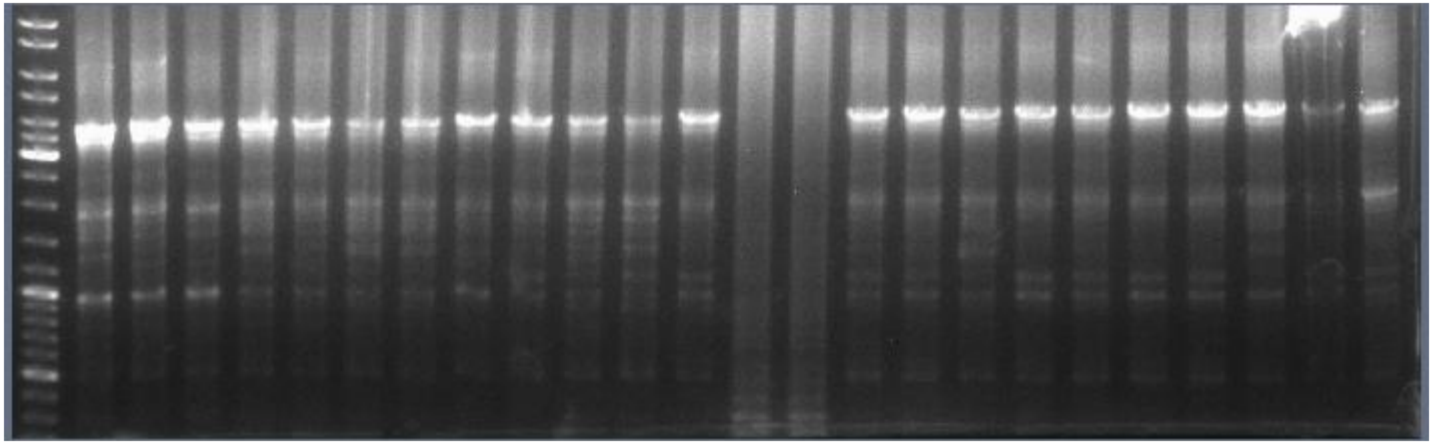

Rep-PCR AB25-AB48

Fig. S6 Rep-PCR of 48 *A . Baumannii* isolates
