# Supplementary figures and images for: Antibacterial properties of Acinetobacter baumanniiphage Abp1 endolysin (PlyAB1)
Source: BMC Infect Dis. 2014 Dec 12;14:681. doi: 10.1186/s12879-014-0681-2 (PMC4274762; doi:10.1186/s12879-014-0681-2)

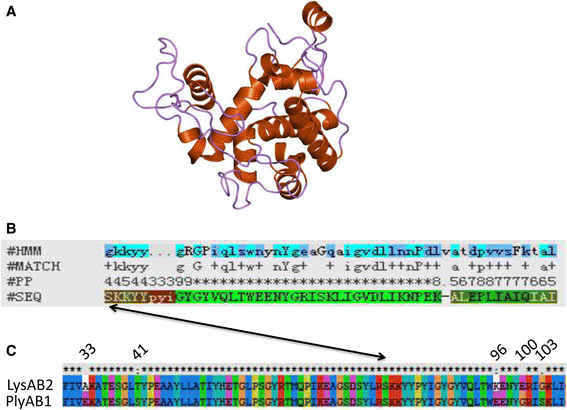

Supplement: Supplementary file 4 — Authors’ original file for figure 1 [file 12879_2014_681_MOESM4_ESM.gif]

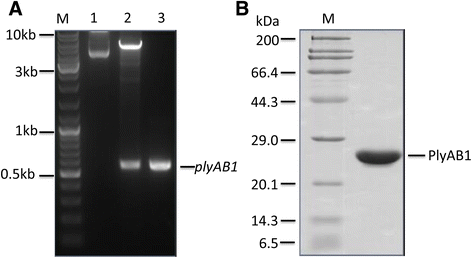

Supplement: Supplementary file 5 — Authors’ original file for figure 2 [file 12879_2014_681_MOESM5_ESM.gif]

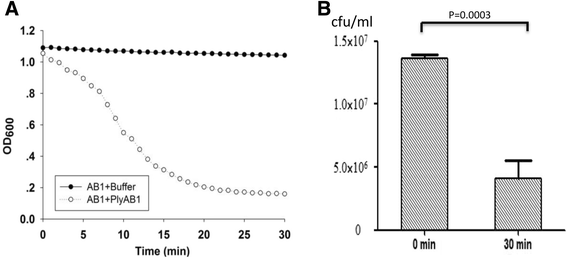

Supplement: Supplementary file 6 — Authors’ original file for figure 3 [file 12879_2014_681_MOESM6_ESM.gif]

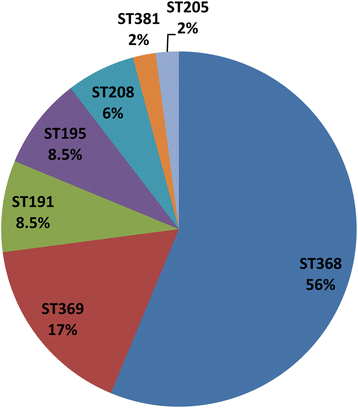

Supplement: Supplementary file 7 — Authors’ original file for figure 4 [file 12879_2014_681_MOESM7_ESM.gif]

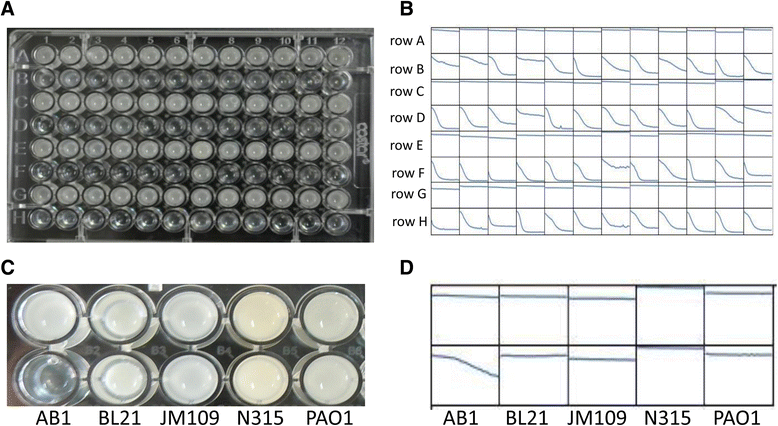

Supplement: Supplementary file 8 — Authors’ original file for figure 5 [file 12879_2014_681_MOESM8_ESM.gif]
